# Correlation between Ocular Manifestations and Their Complications as Opposed to Visual Acuity and Treatment in Behcet's Disease

**DOI:** 10.1155/2013/842673

**Published:** 2013-08-29

**Authors:** Jelena Paovic, Predrag Paovic, Vojislav Sredovic

**Affiliations:** ^1^Department of Ophthalmology, University Clinical Center, Pasterova 2, 11000 Belgrade, Serbia; ^2^Uvea Centar, Center for Diagnostic and Treatment of Uveitis, Kneza od Semberije 14, 11000 Belgrade, Serbia

## Abstract

The aim of this study was to analyze ocular manifestations, their complications, and treatment in a sample of 40 patients with confirmed Behcet's disease. *Results*. Serofibrinous iridocyclitis was the most common form of uveitis (60%). Retinal periphlebitis manifested in 92.5% of cases, and periphlebitis in conjunction with periarteritis was diagnosed in 72.5% of cases. Macular edema was the most frequent complication on the posterior segment (60%) and it correlated with periphlebitis (*P* = 0.45) and periphlebitis associated with periarteritis (*P* = 0.51). Cyclosporine A and corticosteroids were used in the majority of cases (67%). Following six months of therapy, a significant improvement of visual acuity occurred in patients with initial visual acuity >0.5 on both eyes. Level of visual acuity before and after treatment had a strong significant correlation coefficient with various ocular complications. Previously proven significant increase of visual acuity in patients with macular edema depicts effectiveness of treatment in these types of ocular manifestations of Behcet's disease. *Conclusions*. Significant improvement of visual acuity occurred in patients with initial visual acuity >0.5 on both eyes. The highest increase in visual acuity was achieved by laser photocoagulation in combination with triamcinolone acetonide *P* = 0.038 < 0.050.

## 1. Introduction 

Behcet's disease is anidiopathic, multisystem inflammatory chronic relapsing disorder, systemic occlusive vasculitis, which affects veins and arteries of all sizes. Disease is characterized by episodic inflammation which may affect every tissue and organ in the body [1–6]. International Study Group for Behcet's Disease has established diagnostic criteria: ocular lesions, oral aphthosis, and genital aphthosis are each assigned 2 points, while skin lesions, central nervous system involvement, and vascular manifestations 1 point each. The pathergy test, when used, was assigned 1 point. A patient scoring ≥4 points is classified as having Behcet's disease [7]. Behcet's disease has been associated with HLA-B51 phenotype, but the strength of this association varies worldwide [8–18]. 

Ocular features of Behcet's disease manifest as anterior uveitis and retinal vasculitis (both veins and arteries) which result in progressive, irreversible, ischemic damage of the retina and optic nerve. Ocular involvement occurs in approximately 70% of patients and is associated with high risk of blindness [19–22]. Cataract, secondary glaucoma, optic disc atrophy, and macular edema are the most common complications in Behcet's disease leading to decrease of vision [23]. Inflammation of various blood vessels, arteries, veins, and both arteries and veins can lead to diffusion of cystoids macular edema with or without epiretinal membranes. Optical coherent tomography (OCT) and fluorescein angiography are methods used to follow evolution of macular edema [24]. In cases where macular edema is not being treated, it can lead to permanent visual loss [19]. Besides systemic immunosuppressive therapy, use of subtenonial injections of triamcinole acetonide in combination with laser photocoagulation (laser PHC) on retinal periphery, around blood vessels as well as in areas of retinal ischemia, can have an impact on the state of macular edema. Optic disc atrophy is another complication of occlusive retinal vasculitis in Behcet's disease [18, 20]. Progressive loss of vision and blindness from severe occlusive vasculitis and optic atrophy is common despite the aggressive immunosuppressive therapy [25–32]. Over the past couple of years, introduction of biological agents in treatment of Behcet's disease has had positive impact on its prognosis [33–39]. 

This study pays special attention to correlation between visual acuity and ocular manifestations of Behcet's disease and complications of the same. The aim of our study is to verify which procedure is best suited to be used in treatment of uveitis and retinal vasculitis and their various complications and subsequently for improvement of visual acuity. 

## 2. Methods

Ocular manifestations, structural ocular complications, and treatment, as well as incidence rate for loss/improvement in visual acuity, were assessed and presented on a sample of 40 patients with confirmed Behcet's disease. 

## 3. Results

Research sample showed no significant difference in the prevalence of males to females (gender representation was as follows: 21 males or 52.5% versus 19 females or 47.5%, *P* = 0.568 > 0.05). There was also no significant difference between average ages across gender groups (Table 1). 

**Figure 1:**
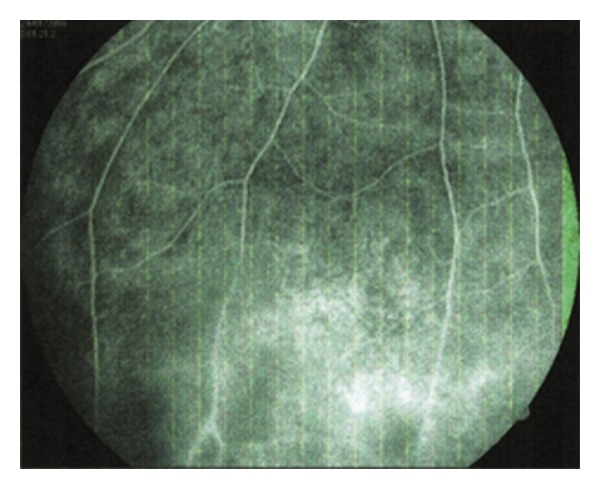
Behcet's disease, retinal periphlebitis.

Dominant form of iridocyclitis as part of ocular manifestations of Behcet's disease was serofibrinous iridocyclitis 60.0% as opposed to the fibrinopurulent one (7.9%). As a consequence to inflammatory changes on the posterior segment of the eye, there was a significant appearance of retinal periphlebitis in Behcet's (92.5%) (Figure 1). 

Periphlebitis in conjunction with periarteritis was diagnosed in a somewhat smaller percentage of individuals (72.5%). Intensity of disease appears to be mild to moderate, but a significant number of individuals (around 30%) has had the more severe form of it. Severe form of uveitis with clinical features, especially thrombosis and haemorrhage in vitreous, manifested in 3 cases. 

**Figure 2:**
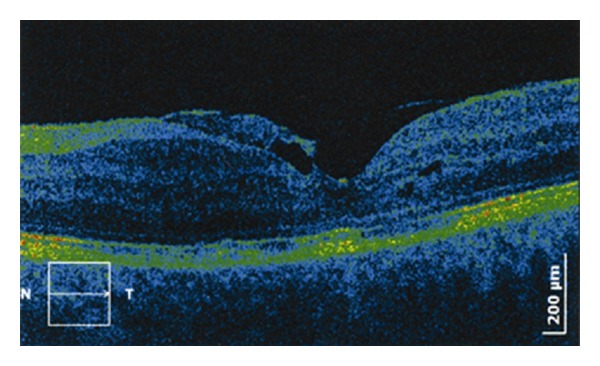
Behcet's disease, macular edema with epiretinal membrane—OCT finding.

In approximately one-third of cases, the most common complication of Behcet's disease of the anterior segment of the eye was complicated cataract. Secondary glaucoma occurred at rates of 17.5%, and macular edema was the most frequent complication of Behcet's disease on the posterior segment of the eye (60%) (Figure 2). 

**Figure 3:**
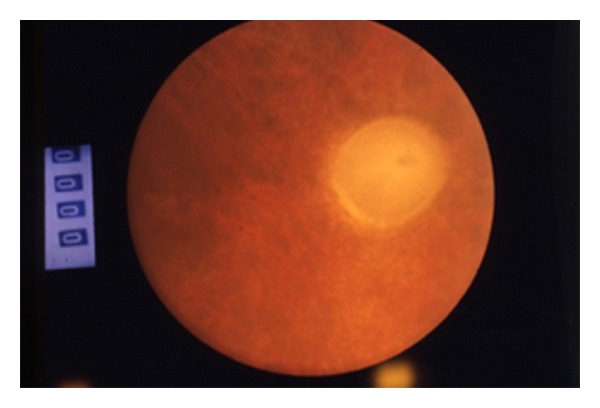
Behcet's disease, disk atrophy.

A rare presence of disc edema and disc atrophy was noted, which is not to say that at a rate of 28.2% and 33.3%, respectively, they held no statistical significance (Figure 3). 

Table 2 presents medical treatment, laser photocoagulation, and surgical methods used in treatment of ocular involvement and complications of Behcet's disease.

 Cyclosporine A (3–5 mg/kg daily) together with prednisolone (20 mg daily) as well as regular monitoring of cyclosporine concentration in blood and tests for liver and kidney functions were part of the therapy applied in the majority of the cases (67.5%) in treatment of ocular inflammation in Behcet's disease. Systemic corticosteroids were used in 17.5%, while other cytostatic medications such as azathioprine (50–150 mg/daily), methotrexate (7.5–25 mg/week), and colchicine of 1.0–2.0 mg/day were used in 15.0% of cases. 

A group of patients which had exhibited improvement of visual acuity mostly underwent treatment comprising cyclosporine A in combination with corticosteroids (67.5%), as well as subconjunctival dexasone injection (25.0%). Besides systemic therapy in treatment of uveitic manifestations on the anterior segment of the eye, in 57.5% of cases, local therapy of corticosteroid drops (dexamethasone 0.1%) and cycloplegic drops (cyclopentolate 0.5–1%) was applied. 

Additional to medicamentous treatment in 57.5% of cases, laser photocoagulation was also used. 

While monitoring intraocular pressure, laser photocoagulation as well as sub-Tenon's injections (4–6 doses) of triamcinolone acetonide (20 mg) was given to a group of 11 (27.5%) patients. Patients who had macular edema and retinal vasculitis was underwent treatment. Surgical methods involved in treatment of uveitic complications were as follows: in 10% of cases, cataract removal was done via phacoemulsification, while phacoemulsification and vitrectomy were performed in 12.5% of patients which exhibited certain retinal complications as well (Table 2). 

Table 3 shows that upon first examination, before treatment was commenced, 60–70% of cases had bilateral visual acuity >0.5. Subsequently, visual acuity of 0.1–0.5 appeared with a frequency of 27% while less frequent were individuals within the interval <0.1 (up to 12.5%). Objective measures of eye conditions used to determine eligibility are visual acuity, visual field, and visual efficiency. We used two of them: visual acuity and visual efficiency. 

Following six months of therapy, data suggests a significant improvement of visual acuity having occurred in those patients with initial visual acuity >0.5 on the right eye (70% to 80%), and slightly less on the left eye (60% to 65%). There appears to be a decrease of visual acuity within the 0.1–0.5 interval (27% to 17.5%, right eye); (27.5% to 17.5%, left eye). Somewhat higher number of cases with lowered values was located within the 0.1 interval (Table 3). 

**Figure 4:**
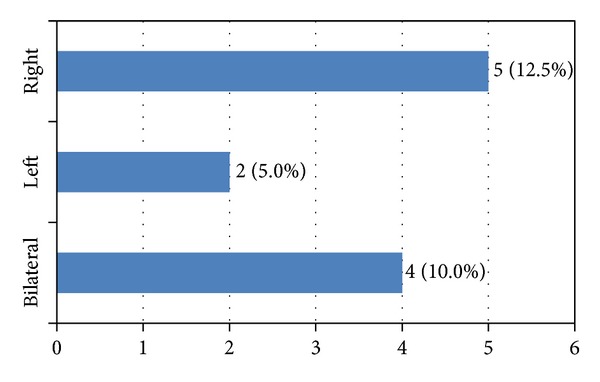
Average visual acuity before and after therapy.

Due to slight difference in bilateral distribution of visual acuity (between left and right eyes), further statistical analysis will be based on average values irrespective of the eye. Comparison of average values of visual acuity had not shown any significant difference, even though previously mentioned value increased the following treatment. Upon initial examination, average visual acuity was 0.68 ± 0.36 irrespective of the eye, and it increased to 0.71 ± 0.36, at follow-up examination. Visual acuity was increased on the right eye in 12.5% of cases on the left eye in 5% of cases, and bilaterally in 10% of cases (Figure 4). 

The majority of medications were introduced to those patients who on average had initially lowered visual acuity. The most frequent improvement of average visual acuity with regard to the initial values occurred in those groups which were treated with cyclosporine A, corticosteroids, or cytostatic drugs (significance level *P* < 0.1). 

Increased visual acuity was noted in patients who underwent laser photocoagulation but this was however not significant at an accepted significance level of *P* < 0.05. 

The highest increase in visual acuity was achieved by laser photocoagulation in combination with triamcinolone acetonide (sub-Tenon's injection) in a group of individuals who had macular edema. Upon further statistical analysis of average visual acuity in this group, it could be concluded that there was a significant increase of visual acuity present on checkup as opposed to the initial assessment averages (0.62 versus 0.67; *P* = 0.038 < 0.050) (Table 4). 

**Figure 5:**
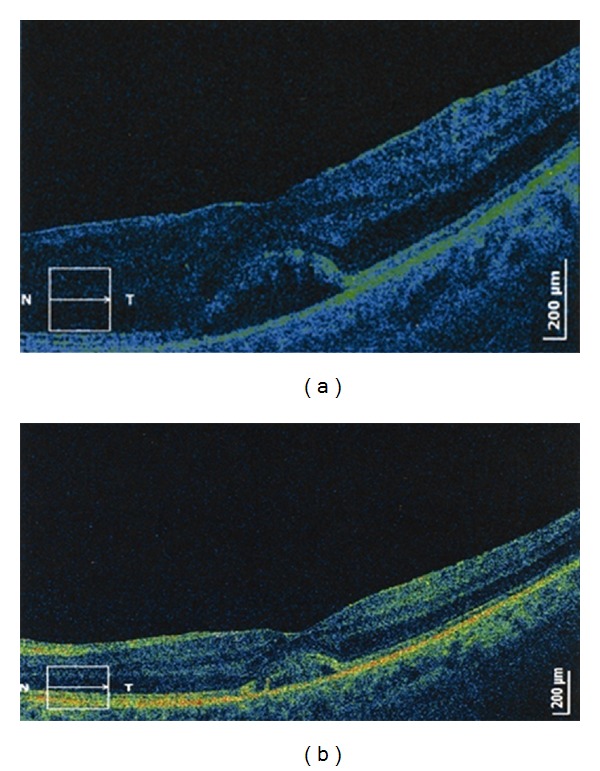
(a) and (b) Behcet's disease, OCT monitoring of macular edema in retinal vasculitis.

OCT method was used in monitoring patients treated for vasculitis and macular edema by laser PHC and sub-Tenon's injections of triamcinolone acetonide (Figures 5(a) and 5(b)). 

Table 5 contains Spearman's correlation coefficients represented within parameters which define ocular manifestations of Behcet's disease and their complications as well as visual acuity before and after treatment was commenced. 

As the preceding analysis has shown, the most important ocular manifestations in Behcet's disease are the presence of retinal periphlebitis including or excluding periarteritis and then macular edema. 

There exists good and statistically significant interdependence of these parameters in Behcet's disease. 

It has been shown that there exists a slightly higher degree of positive correlation (correlation coefficient = 0.51) between central macular edema and the appearance of periphlebitis coupled with periarteritis as primary ocular manifestations. 

High level of significance which exists between appearance of cataract and secondary glaucoma is represented as repercussion of ocular complications on Behcet's disease. 

Levels of visual acuity before and after treatment and a strong significance correlation coefficient show that the appearance of various ocular complications (i.e., cataract, secondary glaucoma, and disc atrophy) is inversely proportional to the degree of visual acuity. 

Data shows that in cases where these complications are progressing, levels of visual acuity are decreasing, which is substantiated by increased negative correlation following therapy, and it is not regarded as a consequence related to treatment of cataract, secondary glaucoma, and disc atrophy. 

Disc atrophy is significantly correlated with the presence of secondary glaucoma and cataract and is defined by positive correlation coefficients in the interval of 0.38–0.52. 

Previously proven significant increase of visual acuity in patients with macular edema depicts effectiveness of treatment in these types of ocular manifestations of Behcet's disease. 

## 4. Discussion

Behcet's disease accounts for up to 20% of cases of endogenous uveitis. The disease is most common along the “Old Silk Route,” which spans the region from Japan and China in the Far East to the Mediterranean Sea, including countries such as Turkey and Iran [40–50]. This is most probably due to association of environmental factors together with the histocompatibility antigen in the same family [8–19]. Eye is the most commonly involved organ in Behcet's disease [2–4, 51]. Approximately in one-fifth of cases, ocular disease may be initial. In more than 50% of the patients, disease is bilateral. In general initial inflammatory process is more anterior, unilateral, but subsequently tends to involve the posterior segment of the eye becoming bilateral. The majority of cases are presented as panuveitis. Fibrinopurulent uveitis was the most common form of anterior uveitis. In our series serofibrinous iridocyclitis was present in 60% of cases. The most common and universal posterior segment findings are vitritis (maybe severe and persistent) and retinal perivasculitis that mainly involve the veins and less frequently the arteries. In our series retinal periphlebitis was in 92.5% of cases and retinal periarteritis associated with periphlebitis was in 72.5% of cases. Severity and number of recurrent of inflammatory exacerbations of the posterior segment determine the extent of permanent ocular structural changes and therefore the resultant rate of irreversible visual loss [19–21]. Severe form of uveitis was present in 30% of cases in our study. The most common complication of ocular Behcet's disease was macular edema which is observed in about half of the patients. In our series macular edema was present in 60% of cases. Cataract formation may also develop during the inflammatory course of the disease or as a consequence of systemic or topical corticosteroid use for the treatment. One-third of our patients developed cataract and in 10% of cases phacoemulsification was performed. Due to retinal complications and complicated cataract phacoemulsification and vitrectomy were performed in 12.5% of cases. At the final period of the disease, the repeated episodes of posterior segment inflammations and complications result in end-stage ocular Behcet's disease with complete blindness. 

Anterior uveitis and cyclitis were treated with topical ophthalmic drops 3–6 times a day (prednisolone 1% or dexamethasone 0.1%). Nonsteroidal anti-inflammatory drugs such as topical indomethacin may prove useful as potentiators of corticosteroid activity. In severe cases however administration should be very frequent for one hour (e.g., every 5 minutes). After 6–8 weeks topical therapy can be discontinued. Topical short-acting mydriatic and cycloplegic agents (tropicamide 1% or cyclopentolate 1%) simultaneously have been added to local corticosteroids twice or three times a day in all cases with anterior uveitis. In cases with severe ciliary spasm, however, the longer acting agents, such as atropine twice daily, had been needed. 

Acute attacks of mild posterior uveitis, cyclitis, and macular edema especially if unilateral, in turn, can be treated with posterior parabulbar sub-Tenon's capsule corticosteroid injection. We have used periocular route for dexasone subconjunctival in 25.0% of cases. Triamcinolon acetonide via anterior parabulbar route in combination with laser photocoagulation was used in 27% of cases. In our group with macular edema, the highest increase in visual acuity was achieved by laser photocoagulation in combination with triamcinolone acetonide (sub-Tenon's injection) (*P* = 0.038 < 0.050). 

Corticosteroids are commonly used as anti-inflammatory agents for the treatment of the majority of Behcet's disease manifestations. Acute and severe disease exacerbations of anterior (poorly responsive to topical/periocular corticosteroid) or posterior, or panuveitis as well as retinitis should be treated with higher dosis of systemic corticosteroid to offer a rapid response. Oral (prednisolone, 1-2 mg/kg/daily or 60–120 mg/daily, given in single morning dose after meals) or intravenous route as pulse methylprednisolone (1 g/daily for three consecutive days) is preferred particularly if bilateral process exists [22, 30]. In our study corticosteroids were used only in 17.5% of cases but in combination with cyclosporine A corticosteroids were used in 67.5% of cases. Cyclosporine A has a more specific effect on the immune system than corticosteroid and cytotoxic drugs [22, 25]. The primary effect of cyclosporine A is the inhibition of T lymphocyte activation. Cyclosporine A is the most rapidly acting agent for acute uveitis at a daily initial dosage of 5 mg/kg, and combined therapy with azathioprine is more effective than monotherapy. The results demonstrated that cyclosporine A alone (5–10 mg/kg/day) or in combination with corticosteroid has a more potent anti-inflammatory effect in an effective treatment of almost every manifestation of Behcet's disease particularly in ocular inflammation, decreasing the frequency and severity of exacerbation with improved visual acuity. 

Besides systemic cyclosporine A and corticosteroid therapy in treatment of severe forms of uveitis, cytostatic drugs such as azathioprine (50–150 mg/daily), methotrexate (7.5–25 mg/week), and colchicine (1-2 mg/daily) are effective in controlling the attacks of posterior ocular inflammation and vasculitis, improving the long-term visual prognoses of the disease with prevention of new eye disease [26–28]. In our study, the most frequent improvement of average visual acuity with regard to the initial values occurred in those groups which were treated with cyclosporine A, corticosteroids, or cytostatic drugs (significance level *P* < 0.1). 

Loss of visual acuity and occurrence of ocular complications were common in patients with ocular inflammations associated with Behcet's disease even with aggressive therapy. In our study the most common ocular manifestation was retinal periphlebitis including or excluding periarteritis and then macular edema. There exists a slightly higher degree of positive correlation between both of them. Significant increase of visual acuity in patients with macular edema depicts effectiveness of treatment in these types of ocular manifestations of Behcet's disease. High level of significance exists between complicated cataract and secondary glaucoma. 

In patients suffering from severe uveitis, biologic therapy can be a breakthrough [31–39]. This kind of therapy for Behcet's disease is not yet registered in our country. 

## Figures and Tables

**Table 1:** Behcet's disease, ocular manifestations and complications (*N* = 40 patients).

	∗	Average age ± SD
Gender		
Male	21 (52.5%)	32.3 ± 13.7
Female	19 (47.5%)	35.0 ± 12.7
Ocular manifestations		
Anterior uveitis		
Serofibrinous iridocyclitis	24 (**60.0%**)	
Fibrinopurulent iridocyclitis	3 (7.9%)	
Cyclitis	7 (17.5%)	
Posterior uveitis		
Retinal periphlebitis	37 (**92.5%)**	
Periphlebitis and periarteritis	29 (**72.5%**)	
Intensity of the inflammatory process		
Severe	12 (30.0%)	
Moderate	9 (22.5%)	
Mild	19 (47.5%)	
Complications of anterior eye segment		
Cataract	13 (32.5%)	
Secondary glaucoma	7 (17.5%)	
Complications of posterior eye segment		
Macular edema	24 (**60.0%**)	
Optic disc edema	11 (28.2%)	
Optic disc atrophy	13 (33.3%)	

∗: number of data (%).

**Table 2:** Medical treatment, laser photocoagulation, and surgical methods used in treatment of ocular involvement and complications of Behcet's disease (*N* = 40).

Treatment of uveitis	
Dexasone subconjunctival	10 (25.0%)
Systemic corticosteroids (prednisolone)	7 (17.5%)
Cyclosporine A and prednisolone	**27 (67.5%) **
Corticosteroid local (dexasone drops)	23 (57.5%)
Cytostatic drugs	6 (15.0%)
Laser PHC	23 ( 57.5%)
Laser PHC and triamcinolone acetonide sub-Tenon's	11 (27.5%)
Surgical treatment of uveitis complications	
Phacoemulsification	4 (10.0%)
Phacoemulsification and vitrectomy	5 (12.5%)

**Table 3:** Behcet's disease, visual acuity (quotient percentage of efficiency) of patients with ocular involvement (*N* = 40).

Visual acuity*	Initial examination		Follow-up examination	
	VEr	VEl	VEr	VEl
<0.1	1 (2.5%)	5 (12.5%)	1 (2.5%)	7 (17.5%)
0.1–0.5	11 (27.0%)	11 (27.5%)	7 (17.5%)	7 (17.5%)
>0.5	28 (70.0%)	24 (60.0%)	32 (80.0%)	26 (65.0%)

*Quotient percentage of efficiency, VE: visual efficiency (r: right, l: left).

**Table 4:** Behcet's disease, average visual acuity efficiency coefficient dependent on treatment and presence of macular edema.

	*N*	Initial examination_1_	Follow-up examination_2_	Sig._1,2_
Treatment				
Dexasone subconjunctival	10	0.4 ± 0.37	0.5 ± 0.39	0.404
Systemic corticosteroids (prednisolone)	7	0.6 ± 0.39	0.7 ± 0.33	0.068^*¥*^
Cyclosporine A and prednisolone	27	0.6 ± 0.37	0.6 ± 0.39	0.667
Corticosteroid (dexasone) drops	23	0.6 ± 0.38	0.6 ± 0.39	0.990
Cytostatic drugs	6	0.5 ± 0.40	0.6 ± 0.42	0.051^*¥*^
Laser PHC	23	0.8 ± 0.33	0.8 ± 0.29	0.825
Macular edema				
Laser PHC and triamcinolone acetonide (sub-Tenon's injections)	11	0.6 ± 0.38	0.7 ± 0.38	0.038*

*Significance *P* < 0.05.

^¥^Significance *P* < 0.10.

**Table 5:** Matrix of correlation parameters: anterior and posterior eye segments, visual acuity (VA before-after treatment).

Parameter	CAT	SGL	CYCL	PPH	PPH & PA	ME	ODE	ODA	VA-b	VA-a
CAT	1.00	0.66*	−0.18	−0.21	0.07	0.24	0.04	0.52*	−0.31*	−0.58*
SGL	0.66*	1.00	−0.21	−0.12	−0.01	0.11	0.00	0.38*	−0.33*	−0.49*
CYCL	−0.18	−0.21	1.00	0.13	−0.31	−0.16	−0.15	−0.05	0.16	0.152
PPH	−0.21	−0.12	0.13	1.00	0.46*	0.45*	0.18	0.20	0.08	0.164
PPH & PA	0.07	−0.01	−0.31	0.46*	1.00	0.51*	0.14	0.32	−0.14	−0.019
ME	0.24	0.11	−0.16	0.45*	0.51*	1.00	0.29	0.26	−0.22	−0.121
ODE	0.04	0.00	−0.15	0.18	0.14	0.29	1.00	0.16	0.16	0.217
ODA	0.52*	0.38*	−0.05	0.20	0.32	0.26	0.16	1.00	−0.33*	−0.36*

CAT: cataract, SGL: secondary glaucoma, CYCL: cyclitis, PPH: periphlebitis, PA: periarteritis, ME: macular edema, ODE: optic disc edema, ODA: optic disc atrophy, VA: visual acuity, b: before, a: after.

*Significant coefficient of correlation *P* < 0.05.
